# The next bastion to be conquered in immunotherapy: microsatellite stable colorectal cancer

**DOI:** 10.3389/fimmu.2023.1298524

**Published:** 2023-12-22

**Authors:** Kai Ding, Pei Mou, Zhe Wang, Shuqing Liu, JinPei Liu, Hao Lu, Ganjun Yu

**Affiliations:** ^1^ Department of Gastroenterology, Changzheng Hospital, Naval Medical University, Shanghai, China; ^2^ Department of Ophthalmology, Changzheng Hospital, Naval Medical University, Shanghai, China; ^3^ Department of General Surgery, Pudong New Area People’s Hospital, Shanghai, China; ^4^ Department of Gastroenterology, Gongli Hospital of Shanghai Pudong New Area, Shanghai, China; ^5^ Department of General Surgery, Changzheng Hospital, Naval Medical University, Shanghai, China; ^6^ Department of Immunology, College of Basic Medicine & National Key Laboratory of Immunity and Inflammation, Naval Medical University, Shanghai, China

**Keywords:** colorectal cancer, tumor microenvironment, microsatellite stability, immune checkpoint inhibitors, immunotherapy

## Abstract

Colorectal cancer (CRC) is the second leading cause of cancer-related deaths worldwide, and its incidence continues to rise, particularly in developing countries. The advent of immune checkpoint inhibitors (ICIs) has represented a significant advancement in CRC treatment. Deficient mismatch repair (dMMR) or high microsatellite instability (MSI-H) serves as a biomarker for immunotherapy, with dMMR/MSI-H CRC exhibiting significantly better response rates to immunotherapy compared to proficient mismatch repair (pMMR)or microsatellite stable (MSS) CRC. While some progress has been made in the treatment of pMMR/MSS CRC in recent years, it remains a challenging issue in clinical practice. The tumor microenvironment (TME) plays a crucial role not only in the development and progression of CRC but also in determining the response to immunotherapy. Understanding the characteristics of the TME in pMMR/MSS CRC could offer new insights to enhance the efficacy of immunotherapy. In this review, we provide an overview of the current research progress on the TME characteristics and advancements in immunotherapy for pMMR/MSS CRC.

## Introduction

Colorectal cancer (CRC) remains a significant global health concern, ranking as the second leading cause of cancer-related deaths worldwide. In 2020, there were approximately 1.9 million new cases of CRC and nearly 1 million deaths attributed to the disease (1). Regrettably, the incidence of CRC continues to rise, particularly in developing countries, and projections indicate that there will be a staggering 2.5 million new cases worldwide by 2035 (2–4). Despite advancements in early screening techniques, such as colonoscopy, which have significantly improved the survival rates of CRC patients, a concerning number of individuals still receive a diagnosis at an advanced stage. Approximately 25% of patients are diagnosed with stage IV CRC, indicating the presence of distant metastasis (5, 6). Furthermore, even patients diagnosed at an early stage can experience disease progression leading to distant metastasis (5, 6). Once CRC has metastasized, the prognosis for patients generally becomes poor (7).

Cancer is a complex disease characterized by the interaction of various cell populations within the tumor microenvironment (TME). In addition to tumor cells, this microenvironment comprises stromal cells, endothelial cells, tumor-associated fibroblasts, immune and inflammatory cells. These diverse cell populations secrete a range of signaling molecules, including growth factors, cytokines, and chemokines, which mutually influence each other and create a microenvironment that supports tumor cell invasion and metastasis (4, 8, 9). Scientists also have discovered that CRC exhibits remarkable heterogeneity, not only in terms of anatomical sites but also in pathology, TME, and drug sensitivity. It is important to note that although the colon is a single organ, there are significant differences between the left and right colon in terms of embryonic origin, anatomical structure, function, molecular characteristics, and TME (10, 11). These differences pose significant challenges in the treatment of CRC. The heterogeneity observed in CRC underscores the need for personalized and targeted therapies that consider the unique characteristics of each patient’s tumor. Understanding the distinct features of CRC subtypes and their associated microenvironments is crucial for developing effective treatment strategies and improving patient outcomes.

Currently, the treatment options for CRC include surgery, chemotherapy, targeted therapy, immunotherapy, and combinations of these approaches. Surgical treatment is the primary choice for early-stage colon cancer, as it can achieve complete resection (R0 resection) and potentially cure the disease. In recent decades, immunotherapy has shown significant progress in the treatment of CRC (12, 13). In 2017, Keytruda received U.S. Food and Drug Administration (FDA) approval for the treatment of solid tumors with the deficient mismatch repair (dMMR) or high microsatellite instability (MSI-H) phenotype, including CRC. Since then, several immune checkpoint inhibitors (ICIs) have been used in clinical practice for CRC treatment. However, it is important to note that only a small fraction of CRC patients possess these specific characteristics. Approximately 85% of CRC patients have the microsatellite stable (MSS) phenotype and do not benefit from ICIs (14). This is primarily due to the cold TME observed in MSS CRC (15). Consequently, the therapeutic response to ICIs in CRC is influenced by differences in the TME. A comprehensive understanding of the immunosuppressive mechanisms within the TME is needed. Effective treatment options for MSS CRC are currently limited, and researchers are actively exploring new strategies. In-depth understanding of the characteristics of the TME in MSS CRC and the development of novel treatment approaches based on this knowledge have become a major focus of current research. Therefore, this article aims to summarize the recent advances in the TME and immunotherapy for MSS CRC, with the goal of providing new ideas and strategies to enhance the clinical efficacy for patients with MSS CRC.

## MMR and MSI

The mismatch repair (MMR) system serves as a protective mechanism in normal human cells, responsible for correcting base mismatches that occur during DNA replication. Mutations or functional defects in the MMR genes can impede the timely and effective repair of mismatched bases, leading to the accumulation of genetic mutations and eventually resulting in tumor formation. Key components of the MMR system include proteins such as MLH1, MSH2, MSH6, and PMS2, as well as genes involved in regulating protein synthesis upstream of these proteins. Mutations or modifications, such as methylation, in MMR genes can cause the loss of MMR proteins, resulting in a condition known as dMMR. Functional impairments in the MMR system can also lead to microsatellite instability (MSI), a condition characterized by the alteration of repetitive DNA sequences.

Microsatellites (MS) are repetitive DNA sequences consisting of short, tandem repeats that are commonly found throughout the genomes of eukaryotic cells. These sequences can be present in various regions of genes, including coding regions (exons), non-coding regions (introns), and gene promoters. They play a crucial role in gene regulation by interacting with specific proteins or influencing DNA structure. The stability of microsatellites during cell division is referred to as MSS. Many important genes involved in growth regulation possess microsatellites in their coding or promoter regions. When the four MMR proteins mentioned earlier (MLH1, MSH2, MSH6, and PMS2) are abnormal, resulting in dMMR, replication errors in these microsatellite repeat regions are not properly corrected. This leads to the accumulation of replication errors, widespread MSI, and ultimately contributes to the development of tumors (16).

MSI is typically associated with a high tumor mutational burden, which is commonly defined as having more than 10 mutations per megabase (Mb). Approximately 15-20% of CRC cases exhibit a high mutational burden phenotype, with the most common cause being defects in DNA MMR. These defects can be inherited, such as in Lynch syndrome, or they can occur sporadically (5). As research progresses, an increasing number of studies have revealed the diversity of gene mutations in CRC. Based on the MSS status, CRC can be classified into two main subtypes: MSS and MSI-H. It has been reported that approximately 85% of CRC cases and 95% of metastatic CRC (mCRC) cases are classified as MSS CRC. In contrast, patients with dMMR or MSI-H CRC exhibit distinct clinical and pathological features. These features include a higher likelihood of occurring in the proximal colon, increased lymphocyte infiltration, and pathological characteristics such as low differentiation, mucinous appearance, or signet ring cell features (17, 18). Furthermore, dMMR/MSI-H CRC patients generally have a better prognosis when distant metastasis is not present (19, 20).

## Characteristics of the TME in CRC

The origin and development of CRC are often intricate processes that involve multiple factors, stages, and steps. Throughout this process, CRC cells and their surrounding environment establish a specific TME, and the interaction and co-evolution of various components within the TME contribute to its occurrence and progression. Notably, CRC with different microsatellite statuses exhibit significant differences in the TME (21).

**Figure 1:**
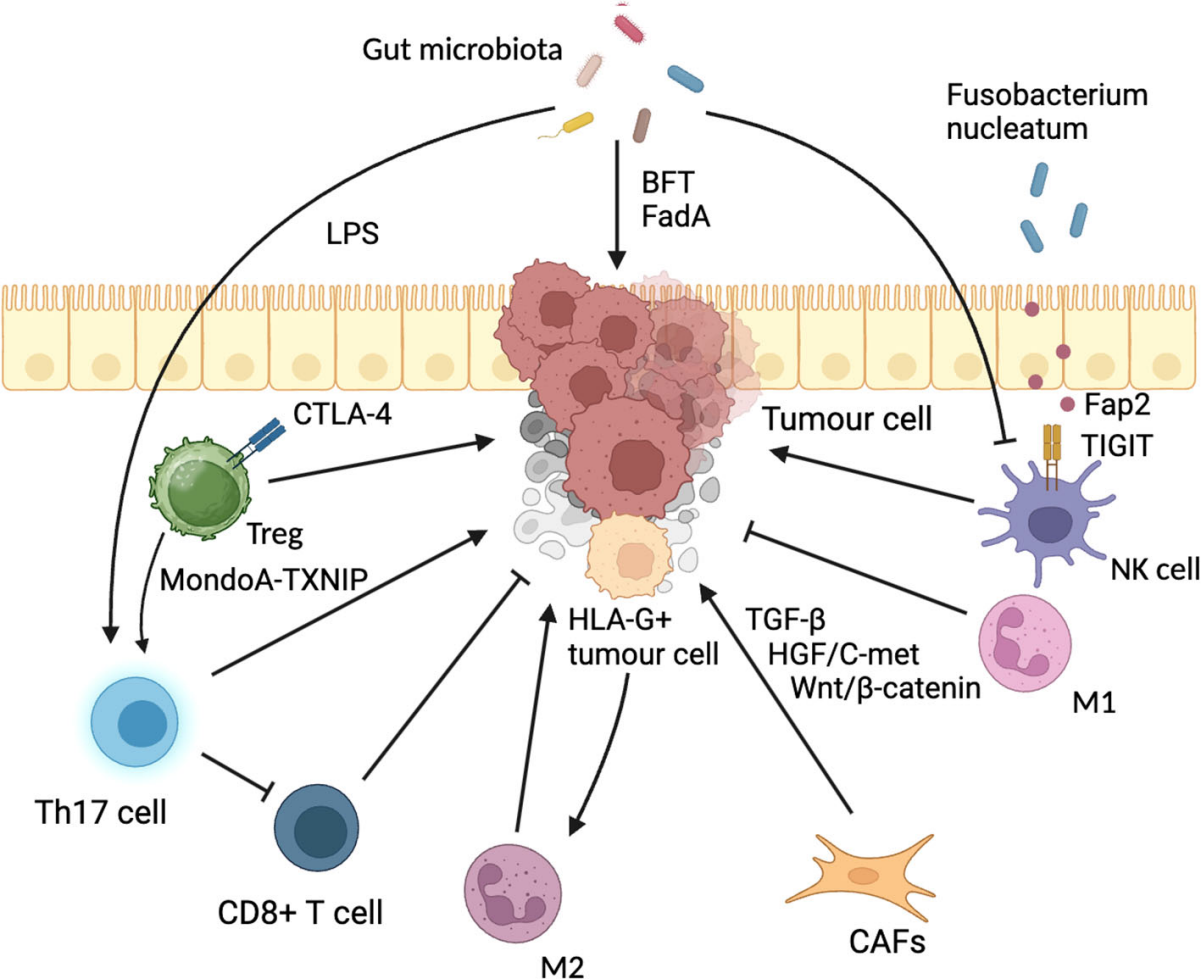
Tumor microenvironment in CRC. DC, dendritic cell; IFN-DC, Inflammatory DC; Treg, regulatory T cell; NK cell, Natural killer cell; Th17, T helper cell 17; LPS, lipopolysaccharide; TGF-β, transforming growth factor-beta; CTLA-4, Cytotoxic T Lymphocyte-Associated Antigen-4; GM-CSF, granulocyte-macrophage colony-stimulating factor; CAFs, cancer-associated fibroblasts; HLA-G, human leucocyte antigen-G; FadA, Fusobacterium adhesin A; BFT, Bacteroides fragilis toxin; TIGIT, T cell immunoglobulin and ITIM domain.

The TME encompasses the dynamic changes that occur in the surrounding tissues during the development and progression of a tumor, leading to the formation of a complex tumor stroma. It involves various cell types, such as immune cells, endothelial cells, fibroblasts, and others, as well as extracellular components like cytokines, growth factors, hormones, and the extracellular matrix, all of which surround the tumor cells (22). Among these components, immune cells play a particularly crucial role in shaping the microenvironment. T lymphocytes, tumor-associated macrophages (TAMs), natural killer (NK) cells, dendritic cells (DCs), neutrophils, and various factors interact with each other, collectively forming the CRC immune TME, which significantly influences tumor development and prognosis (23). Additionally, the gut microbiota also participates in modulating the TME, and the intricate network of interactions among immune cells, tumor cells, microbiota, various factors, and metabolic products constitutes the intra-tumoral network (24, 25) (**Figure 1**).

In terms of the immune microenvironment, MSI-H CRC is predominantly characterized as an immune-inflammatory type, while MSS CRC is mostly categorized as immune-exempt or immune-desert types (26). Numerous studies have demonstrated that the expression of Th1, Th2, CD8+ cytotoxic cells, follicular helper T cells, and T cell markers is significantly higher in MSI-H CRC compared to MSS CRC. Furthermore, MSI-H patients exhibit a higher tumor mutation burden, frameshift/insertion-deletion mutations, and a greater number of tumor neoantigens compared to MSS patients. The lower mutation burden and fewer neoantigens in MSS CRC may limit recognition and attack by the immune system, resulting in reduced sensitivity to immune therapy. Hence, it can be inferred that immune cell infiltration plays a crucial role in the differential MMR status of different tumors, leading to the classification of CRC as “cold” or “hot.” A comprehensive analysis of the changes in different components of the TME is essential for understanding tumor initiation and progression, suppression of immune response, and determining the suboptimal response to immunotherapy.

## Immune cells in TME

Tumor-infiltrating lymphocytes (TILs) are a diverse group of immune cells that reside within the TME. They include cytotoxic T cells, helper T cells, regulatory T cells, B cells, and others. These TILs play a crucial role in immune responses, thereby influencing tumor growth and treatment response. However, TILs exhibit a dual nature: they can recognize and eliminate tumor cells, exerting anti-tumor effects, such as cytotoxic T cells (CTLs) and NK cells. On the other hand, certain subsets of TILs present in the tumor environment, such as regulatory T cells and TAMs, may inhibit immune responses and promote immune evasion by the tumor.

Regulatory T cells (Tregs) are a specialized subset of immune cells that infiltrate the TME and contribute to the establishment of an immunosuppressive milieu. Targeting Tregs has emerged as an important approach in tumor immunotherapy. In CRC, Tregs experience various metabolic stresses, including hypoxia, nutrient deprivation, and acidic environments, which lead to heterogeneous metabolic patterns and functional characteristics. Tong et al. discovered that Tregs infiltrating CRC exhibit enhanced glycolytic activity. This metabolic shift is mediated by the downregulation of the MondoA-TXNIP transcriptional regulatory axis, resulting in increased expression and membrane localization of the glucose transporter Glut1. Consequently, a Th17-like Treg cell subset with a glycolytic metabolism is induced. This metabolic alteration weakens the immunosuppressive function of Tregs, promotes Th17-type inflammation, and inhibits the anti-tumor function of CD8+ T cells, thereby promoting CRC development (22). Additionally, Tregs express the immune checkpoint receptor cytotoxic T lymphocyte-associated antigen-4 (CTLA-4), which contributes to tumor immune tolerance. The m6a-modified circQSOX1 promotes PGAM1 expression by sequestering miR-326 and miR-330-5p, thus activating glycolysis and impairing the response to anti-CTLA-4 therapy, ultimately leading to immune evasion in CRC. Therefore, a combination therapy targeting sh-circQSOX1 and anti-CTLA-4 may represent a strategy to overcome Treg cell-mediated immunotherapy resistance in CRC (27).

TAMs are a type of macrophage that infiltrate tumor tissues and play a significant role in the TME. TAMs can differentiate into two distinct polarization states: M1 macrophages, which possess phagocytic and tumoricidal activities, and M2 macrophages, which promote angiogenesis, tissue remodeling, wound healing, and tumor progression (28). In the context of colon cancer liver metastasis, M2 macrophages secrete various cytokines and chemokines that contribute to the process. Recent studies utilizing spatial transcriptomics and single-cell sequencing have shed light on the interaction between HLA-G+ cancer cells and SPP1+ macrophages in the immune microenvironment at the invasive front of CRC. HLA-G+ cancer cells can induce macrophages to polarize towards an M2 phenotype, characterized by the expression of SPP1 (osteopontin). This polarization inhibits the anti-tumor immune response within the microenvironment (29).

## Cancer-associated fibroblasts

Mesenchymal stem cells (MSCs) and fibroblast-like cells have long been recognized as important stromal cell types involved in regulating tumor initiation and cancer progression. Among these, CAFs are the predominant stromal cells in the TME and are often enriched in CRC, where their presence is associated with prognosis (30). CAFs play diverse roles in tumor biology, including extracellular matrix remodeling, immune response suppression, angiogenesis promotion, metabolism regulation, and maintenance of tumor cell stemness. They contribute to various biological processes such as inhibition of apoptosis, tumor proliferation, invasion, migration, immune evasion, and drug resistance (31–34). CAFs can differentiate from various cell types, and recent studies have revealed distributional differences of different fibroblast subtypes in tumor tissues. For example, FAP+ fibroblasts, proliferative fibroblasts, and pericytes are significantly enriched in tumor tissues, while NT5E+ fibroblasts, FGFR2+ fibroblasts, ICAM1- terminal fibroblasts, and MFAP5+ myofibroblasts are enriched in adjacent normal tissues. The interaction between FAP+ fibroblasts and SPP1+ macrophages contribute to the formation of a desmoplastic TME, which hinders lymphocyte infiltration and may lead to resistance to tumor immunotherapy. CAFs also play a role in colon cancer liver metastasis through signaling pathways such as TGF-β, Wnt/β-catenin, HGF/C-met, and various cytokines (35).

## The gut microbiota

The gut microbiota, which is the most important and complex ecosystem in the human body, plays a crucial role in the TME (36, 37). The composition of the predominant intestinal microbiota varies across different anatomical regions of the gastrointestinal tract. Under normal circumstances, the structure, diversity, and abundance of gut bacteria remain relatively stable, maintaining a balanced state with the host and external environment. However, dysbiosis of the gut microbiota has been associated with various diseases and tumors (38). Studies have revealed that patients with CRC exhibit a significant increase in bacteria such as Clostridium, Enterococcaceae, and Enterococcus in their fecal samples, while bacteria such as Bacteroides and Lactobacillus are relatively decreased. These findings highlight the potential role of the gut microbiota in CRC development and progression (39–41).

The gut microbiota can directly contribute to the development of CRC through various mechanisms. For example, at the site of intestinal tumors, the genus Escherichia secretes colibactin, a genotoxic peptide toxin, while the genus Bacteroides secretes reactive oxygen species, both of which can induce genomic instability and promote genomic mutations (42). Indirectly, the gut microbiota can promote tumorigenesis by inducing inflammation or exerting immunosuppressive effects (43). Specific bacteria within the gut microbiota have been identified for their role in CRC development. For instance, Enterotoxigenic Bacteroides fragilis (ETBF) produces Bacteroides fragilis toxin (BFT), and Fusobacterium nucleatum produces Fusobacterium adhesin A (FadA). These bacteria can activate various signaling pathways such as Wnt/β-catenin, MAPK, NF-κB, and STAT3, leading to the production of cytokines (IL-17, IL-23, IL-6, IL-1, etc.) and promoting intestinal mucosal inflammatory responses (44–47). F. nucleatum also expresses the Fap2 protein on its surface, which interacts with T cell immunoglobulin and ITIM domain (TIGIT), inhibiting the cytotoxicity of NK cells and protecting tumor cells from infiltration by lymphocytes and T cells (48, 49). Moreover, high levels of F. nucleatum in tumor tissue have been associated with lower infiltration by T cells, resulting in a reduction of the antitumor immune response (50, 51).

In addition, non-toxinogenic strains of Fusobacterium can activate the β-catenin/CCL28 axis through bile salt hydrolase (BSH), leading to an increase in immunosuppressive CD25+FOXP3+ regulatory T cells (Treg) within the tumor. This promotes the progression of CRC (52). Furthermore, the bacterial metabolite lipopolysaccharide (LPS) can activate the “intestinal epithelial CCL2-monocytic macrophage-Th17 cell” regulatory network, leading to the specific enrichment of monocytic macrophages in the early stages of inflammation-associated tumorigenesis. These macrophages secrete inflammatory factors that promote the development of intestinal tumors (53).

The gut microbiota exerts its influence on the host through various mechanisms, including the regulation of metabolite levels in the host’s serum, immune activity, intestinal permeability, and even direct effects such as DNA damage, hepatotoxicity, and carcinogenesis. Metabolites produced by the gut microbiota, such as short-chain fatty acids (e.g., butyrate, propionate), succinate, aromatic amino acid metabolites, and secondary bile acids, have been implicated in these processes (54). Moreover, Fusobacterium nucleatum has been found to promote chemotherapy resistance in CRC by regulating autophagy, which contributes to CRC recurrence and poor prognosis (54). Autophagy is a cellular process involved in the degradation and recycling of cellular components, and dysregulation of autophagy can impact cancer development and treatment response. In the context of CRC, changes in the TME have been observed in relation to innate lymphoid cells (ILCs). ILCs are a family of tissue-resident lymphocytes that play a crucial role in regulating host-microbiota interactions at the mucosal barrier. In a healthy gut, the “dialogue” between ILC3 and T cells is essential for maintaining immune homeostasis and shaping the microbiota in a way that supports type 1 immunity. However, studies have shown alterations in type 3 innate lymphoid cells (ILC3) within the TME of CRC patients. These alterations include a decrease in frequency, increased plasticity, and imbalanced interaction with T cells, all of which contribute to CRC progression or resistance to ICIs (55). Understanding these interactions between ILCs, T cells, and the gut microbiota in the TME is crucial for developing effective therapeutic strategies for CRC.

Based on the information provided, it is evident that the gut microbiota plays a significant role in the CRC TME. The interactions between the gut microbiota and immune cells create a network within the tumor, influencing the formation of the tumor immune microenvironment. This understanding serves as an important foundation for exploring interventions targeting the gut microbiota in the development and progression of CRC. However, the specific correlation and impact of gut microbiota characteristics on different types of CRC, such as those with dMMR or proficient mismatch repair (pMMR), have not been extensively studied. It remains unclear whether the gut microbiota can serve as a biological target for predicting treatment efficacy and prognosis in CRC. Further research is needed to investigate these aspects and determine the potential of gut microbiota-based interventions in personalized treatment strategies for CRC.

## Advance of cancer immunotherapy in MSS CRC

### ICIs

In 2017, Keytruda (Pembrolizumab) received FDA approval for the treatment of solid tumors with dMMR or MSI-H, leading to significant survival improvements in MSI-H CRC patients. However, MSI-H patients account for only 5% of mCRC cases, while the majority of patients have MSS tumors (56). Most MSS CRC patients still rely on chemotherapy as the primary treatment, and effective therapy options for this population are limited. Clinical studies have demonstrated that single-agent immunotherapy is generally ineffective for MSS patients (57–60). Therefore, there is a need for further research and breakthroughs in immunotherapy for MSS CRC. Currently, researchers are actively investigating MSS CRC patients and striving to improve clinical efficacy for this large proportion of CRC patients. The key strategy is to overcome the treatment challenge of MSS colon cancer through combination therapy, with the aim of transforming MSS “cold” tumors into “hot” tumors similar to MSI-H tumors (61). Recent years have seen significant efforts in clinical research focused on MSS CRC, and representative findings from these studies are summarized in **Table 1**. These studies aim to identify novel therapeutic approaches and combination strategies to enhance the immune response and improve outcomes for MSS CRC patients.

**Table 1:** Representative clinical research findings on MSS CRC in recent years.

• Year/study	• Treatment	• Population	• Important findings
2020/REGONIVO, EPOC1603 (an open-label, dose-escalation, and dose-expansion phase Ib trial) (62)	Regorafenib+Nivolumab	• N=50. (25 each with GC and CRC) • MSI-H: 1 CRC; MSS/pMMR: 49.	• ORR: 40% (20/50); 44% (11/25) in GC; 36% (9/25) in CRC. • mPFS: 5.6 months (95% CI, 2.7-10.4) inGC; 7.9 months (95% CI, 2.9-NR) in CRC.
2021/REGOMUNE (a single-arm, open-label, phase II trial) (63)	Avelumab + Regorafenib	• N=48. • MSS advanced or mCRC patients and received at least one previous line of systemic treatment. • Among them, 43 assessable for efficacy.	• ORR: 0%; SD 53.5% (23/43); PD 39.5% (17/43). • mPFS: 3.6 months [95% CI, 1.8-5.4]; • mOS:10.8 months (95% CI, 5.9-NA). • High baseline infiltration by TAM was significantly associated with adverse PFS (1.8 vs. 3.7 months; *P* = 0.002) and OS (3.7 months vs. not reached; *P* = 0.002). • Patients with increased infiltration by CD8+ T cell at cycle 2 Day 1 compared to baseline had significantly better PFS (3.7 vs 2.3 months, *P* =0.035) and OS (NR vs 4.3 months, *P* =0.03).
2021/ EPOC1704 (an open-label, dose-finding, and expansion phase Ib trial) (64)	Nivolumab+TAS-116 (an Oral HSP90 Inhibitor)	• N=44. • advanced or metastatic solid tumors refractory to or intolerant of standard chemotherapy. • MSI-H: 1 CRC; MSS/pMMR: 36 (28 CRC+8 GC).	• ORR: 16% (95% CI, 5–36) in MSS CRC without prior anti–PD-1/PD-L1 Ab. • The median duration of response was 8.6 months (95% CI, 2.9-NR) in 4 MSS CRC. • mPFS: 3.2 months (95% CI, 2.8-4.4) in MSS CRC without prior anti–PD-1/PD-L1 Ab. The PFS rate at 4 months or 6 months was 36.0% or 24.0% in MSS CRC without prior anti–PD-1/PD-L1 Ab. • mOS: 13.5 months (95% CI, 8.2–15.1) in MSS CRC without prior anti–PD-1/PD-L1 Ab.
2021/LEAP-005 (a phase II multicohort study) (65)	Pembrolizumab (anti-PD-1)+Lenvatinib	• N=32. • metastatic and/or unresectable CRC, non–MSI-H/pMMR tumor per local determination, previous treatment with oxaliplatin and irinotecan in separate lines of therapy	• ORR(CR+PR): 22% (95%CI, 9-40), DCR(CR+PR+SD) : 47% (95%CI, 29-65); • mPF: 2.3 months (95%CI, 2.0-5.2); • mOS: 7.5 months (95%CI, 3.9-NR)
2022/ PICCASSO (a phase I trial) (66)	Pembrolizumab+CCR5 inhibitor	• N=20. • refractory pMMR CRC.	• Of the 20 patients, 1 not evaluable, 1 PR, 18 PD. • mPFS was only 9 weeks. • mOS was 9 months.
2022/AtezoTRIBE (a multicentre, open-label, randomised, controlled, phase II trial) (67)	Control group: FOLFOXIRI+Bevacizumab; N=73; Atezolizumab group: FOLFOXIRI+Bevacizumab+Atezolizumab (anti-PD-L1); N=145;	• N=218. • unresectable, previously untreated mCRC. • Tumor MMR status was successfully tested in 212 (97%) of 218 patients, and dMMR was detected in 13 (6%) patients (eight in the atezolizumab group and five in the control group).	• Compared with chemotherapy and targeted therapy, the addition of immunotherapy significantly improved mPFS (11.5 vs. 13.1 months, HR 0.69, 95% CI 0.56-0.85, P=0.012). • After excluding dMMR patients, the analysis of the pMMR subgroup also showed an improvement trend in mPFS (11.4 vs. 12.9 months, HR 0.78, 95% CI 0.62-0.97, P=0.071).
2022/ A real-world study (68)	RS group: Regorafenib+ Sintilimab; N=42 FS group: Fruquintinib+ Sintilimab; N=30; third-line or above therapy	• N=72. • Patients with MSS mCRC who have failed from prior treatment.	• In the general population, the ORR and DCR were 13.9% and 70.8%, and the mPFS and mOS was 4.2(95% CI, 2.9-5.5) and 10.5 (95% CI,8.6-12.4) months, respectively. • There were no statistically significant differences between RS and FS group in PFS (3.5(2.2-4.8) vs. 5.5(3.5-7.5) months, P=0.434) and OS (11.0(7.0-15.0) vs. 10.5(3.8-17.2) months, P=0.486). • Subgroup analysis suggested that patients without liver metastasis responded well to this combination regimen (ORR: 21.4% vs. 9.1%) and obtained better OS (26(8.8-43.2) vs. 10.0(7.4-12.6) months, P=0.016).
2023/ NEST-1 (investigator-initiated trial, NCT05571293) (69)	Botensilimab (anti-CTLA-4)+Balstilimab (anti-PD-1)	• N=70. • locally advanced pMMR CRC.	• The ORR was 23%, and 11/16 were still being followed up at the time of reporting. • Remission was observed in all patients without liver metastasis. Importantly, 81% of patients without liver metastasis were alive at 12 months.
2023/ MEDITREME (a phase Ib/II trial) (70)	Durvalumab (anti-PD-L1)+Tremelimumab (anti-CTLA-4)+FOLFOX	• N=57. • RAS-mutant untreated and unresectable mCRC. • MSI: 3; MSS: 48. • Only the 48 MSS tumors were included in the eligible population for efficacy analyses.	• ORR: 64.5% (31/48); PR: 52% (25/48); CR: 12.5% (6/48). DCR (CR+PR+SD): 93.7%. • 3-month PFS: 90.7% (95% confidence interval (CI): 79.2–96%). Six-month, 12-month and 24-month PFS was, respectively, 60.4% (95% CI, 45.2–72.6%), 26.9% (95% CI, 15.3–39.9%) and 6.7% (95% CI, 1.8–16.5 %). mPFS was 8.2 months (95% CI, 5.9–8.6). • OS at 6 months, 12 months and 24 months, was respectively, 95.8% (95% CI, 84.3–98.9%), 81.1% (95% CI, 66.8–89.7%) and 57.6% (95% CI, 42.3–70.2%). mOS was not reached.
2023/VOLTAGE-A (Investigator-initiated clinical trial, phase Ib/II study) (71)	Preoperative chemoradiotherapy+ Nivolumab+ radical surgery	• N=42. • 37 resectable MSS mCRC.	• With a median follow-up of 44.8 months (range, 25.7-58.9), the 3-year RFS and 3-year OS rates were respectively 79.5% and 97.4% in MSS, and 100% in MSI-H. • Of the MSS, those with pCR, cCR according to the MSKCC criteria, high PD-L1 expression (TPS ≥1%), and CD8/eTreg ratios of ≥2.5 had a trend of better 3-year RFS and OS than those without.
2023/BBCAPX (a randomized, open-label, multicentric study) (72)	CAPEOX+Bevacizumab; CAPEOX+ Bevacizumab +Sintilimab (anti-PD-1)	• N=25. • patients with untreated, RAS-mutant, MSS, unresectable mCRC.	• CR: 8.0% (2/25); PR: 76.0% (19/25); SD: 16.0% (4/25). • Patients with liver or lung metastasis had a higher ORR (93.3% and 100%, respectively) compared to the overall ORR (84.0%). • DCR (CR+PR+SD): 100%. • mPFS has not reached.
2023/ NCT03903705 (phase Ib/II, open-label, multi-centre, multi-cohort dose-escalation and dose-expansion study) (73)	Fruquintinib+Sintilimab	• N=44. • Among 44, 37 patients with mCRC in the dose-expansion phase, 25 (67.6%) were identified as pMMR, and MMR status was not available for the other 12 (32.4%) patients.	• In pooled mCRC analysis, the ORR was 23.8% (95% CI, 8.2–47.2), mPFS was 6.9 months (95% CI,5.4–8.3), and mOS was 14.8 months (95% CI 8.8–NR); • In mCRC patients with pMMR, mPFS and mOS were 20.0% (95% CI, 4.3–48.1), 6.9 months (95% CI, 4.8–10.1), and 20.0 months (95% CI 8.1–NR), respectively.

95% CI, 95% confidence interval; Ab, antibody; cCRclinical complete response; CR, complete response; DCR, disease control rate; dMMR, deficient mismatch repair; GC, gastric cancer; mCRC, metastatic colorectal cancer; mOS, median overall survival; mPFS, median progression-free survival; MSI-H, microsatellite instability-high; MSKCC, Memorial Sloan Kettering Cancer Center; MSS microsatellite stability; NR, not reached; ORR, objective response rate; pCR, pathological complete response; pMMR, proficient mismatch repair; PR, partial response; RFS, relapse-free survival; SD, stable disease; TAM, tumor-associated macrophages; TPS, tumor proportion score.

#### Immunotherapy plus chemotherapy

Most studies indicate that chemotherapy drugs, such as fluorouracil (FU), platinum compounds, alkylating agents, and taxanes, have the potential to improve the tumor immune microenvironment through various mechanisms. These mechanisms include inducing immunogenic cell death, increasing the expression of major histocompatibility complex class I (MHC-I) molecules, releasing tumor cell neoantigens, modulating immune suppressive cells, and upregulating programmed cell death ligand 1 (PD-L1) expression on tumor cells. However, it’s important to note that the immunomodulatory effects of chemotherapy can vary significantly depending on factors such as the specific chemotherapy agent, dosage, administration method, and sequencing (74). Different chemotherapy drugs may have distinct impacts on the immune system and the TME. For example, some chemotherapeutic agents may enhance the immune response by promoting the release of tumor-associated antigens and activating immune cells, while others may have immunosuppressive effects. The dosage and administration schedule of chemotherapy can also influence its immunomodulatory effects.

Additionally, the timing and sequencing of chemotherapy in combination with immunotherapy or other treatments may impact the overall therapeutic outcome. Further research is needed to better understand the complex interactions between chemotherapy and the immune system, as well as to optimize treatment regimens that combine chemotherapy with immunotherapy or other immune-modulating approaches. This knowledge will help guide the development of more effective treatment strategies for CRC and other malignancies.

Some researchers have hypothesized that short-term treatment with Oxaliplatin-based chemotherapy, such as FLOX (Fluorouracil, Leucovorin, and Oxaliplatin), can potentially transform MSS mCRC into an immunogenic state. This transformation may allow previously untreated patients with unresectable mCRC to achieve durable disease control when combined with ICIs. To test this hypothesis, several studies have evaluated the efficacy and safety of ICIs in combination with FLOX, mFOLFOX7 (modified FOLFOX7), or FOLFIRI (Folinic acid, Fluorouracil, and Irinotecan) regimens specifically for MSS CRC patients (75–77).

However, the results of these studies have not shown significant advantages compared to previous first- or second-line chemotherapy regimens combined with targeted therapies. Despite the potential benefits of combining chemotherapy and immunotherapy, the outcomes have not met expectations in MSS CRC patients. Moving forward, further exploration may focus on triplet therapy, which combines chemotherapy, targeted therapy, and immunotherapy, or other combination treatment regimens based on immunotherapy, in order to improve the clinical outcomes for MSS CRC patients. Ongoing research aims to identify more effective strategies to enhance the immune response and achieve better treatment responses in this patient population.

#### Immunotherapy plus radiotherapy

In theory, radiotherapy and immunotherapy can have a synergistic effect. Radiotherapy can release tumor antigens, leading to increased T-cell infiltration into the tumor, upregulation of PD-L1 expression in tumor tissue, and enhanced secretion of anti-tumor cytokines derived from T cells. Simultaneously, immunotherapy helps to alleviate T-cell suppression and enhance the tumor-killing effect of radiotherapy. Furthermore, the combination of radiotherapy and immunotherapy can also induce a “distant effect,” meaning it can target tumors outside the radiation field (78–80).

Based on this mechanism, a phase II study investigated the combination of Nivolumab and Ipilimumab with focal radiotherapy in the treatment of 40 cases of MSS mCRC (81). The results demonstrated an objective response rate (ORR) of 12.5% and a disease control rate (DCR) of 29.2%. Excitingly, the median overall survival (OS) of patients with DCR reached 15.8 months. Another single-arm, non-randomized, phase II clinical trial conducted by Ting et al. (NCT03104439) used Nivolumab + Ipilimumab + hypofractionated radiotherapy to treat 40 patients with MSS mCRC (82). The results of this study showed that the combination of radiotherapy and immunotherapy can prolong the OS of patients with MSS mCRC and improve the response rate of ICIs. This provides a new treatment strategy for patients with MSS mCRC.

Currently, there are limited clinical studies on the combination of immunotherapy and radiotherapy, and further exploration is expected in the future. Additionally, determining the optimal combination sequence and treatment doses of radiotherapy and immunotherapy to achieve the best therapeutic effect is an area that requires further investigation.

#### Immunotherapy plus anti-angiogenic therapy

Preclinical studies have demonstrated that inhibitors targeting vascular endothelial growth factor (VEGF) and its receptor (VEGFR) can reduce tumor angiogenesis and normalize blood vessels. This normalization of blood vessels increases oxygen supply, enhances the delivery of anti-tumor drugs, and promotes the infiltration of effector T cells into the tumor. Consequently, it efficiently activates and initiates T cell responses while reducing the infiltration of immunosuppressive cells like M2 tumor-associated macrophages (TAMs-M2) and regulatory T cells. Therefore, VEGF/VEGFR inhibitors have a synergistic effect when combined with immunotherapy (83).

Several prospective single-arm studies have investigated the efficacy of ICIs in combination with tyrosine kinase inhibitors (TKIs) as third-line treatment for MSS mCRC. These studies include combinations such as Regorafenib with PD-1/PD-L1 antibodies [REGONIVO, REGOMUNE (84), REGOTORI (85)], Fruquintinib with a PD-1 antibody [FRESCO (86)], and Lenvatinib with Pembrolizumab (LEAP-005). However, the clinical studies mentioned above, which explored the combination of immunotherapy and TKIs with anti-angiogenic effects, have not yielded satisfactory efficacy. Furthermore, most of these studies were not randomized controlled trials (RCTs), so their conclusions require further confirmation. The IMblaze370 study (87) compared the efficacy of a PD-L1 inhibitor in combination with a MEK inhibitor (Atezolizumab plus Cobimetinib) to standard regorafenib as a third-line treatment. The long-term follow-up results showed no significant difference in OS between the treatment groups, but an increase in adverse reactions was observed (88).

Previous studies have demonstrated that Cetuximab, an EGFR inhibitor, has direct tumor-killing effects and can induce immune effects through dependent cell-mediated cytotoxicity as an IgG1 monoclonal antibody. It recruits anti-EGFR T cells and CD8+/CD3+ T cells. Additionally, Cetuximab can enhance the expression of PD-L1 on tumor cells, leading to immune suppression and suggesting a potential synergistic effect with PD-1 antibodies (89–91).

Based on this theory, the CAVE study evaluated the efficacy of the combination of cetuximab and avelumab in patients with RAS wild-type mCRC who had previously failed at least two lines of standard treatment. This study included 71 MSS patients and found a median OS of 11.6 months, a median progression-free survival (PFS) of 3.6 months, and an ORR of 8.5% (92). Another phase IIa study compared the effect of Avelumab plus Cetuximab plus chemotherapy (FOLFOX) in treatment-naive patients with RAS/BRAF wild-type mCRC (93). The results showed a 1-year PFS rate of 40%, a median PFS of 11.1 months, and an early tumor shrinkage rate (ETS) of 81%. However, the At533PD study (Capecitabine plus Bevacizumab with Atezolizumab or placebo for refractory advanced CRC patients) (94) and the MODUL study (5-FU plus Bevacizumab plus Atezolizumab for maintenance treatment in patients with stable disease after first-line therapy) (95) did not demonstrate improved PFS and OS.

Overall, the combination strategy of immunotherapy with anti-angiogenic treatment has yielded disappointing results. The specific mechanisms underlying the differential efficacy of combination therapy with anti-angiogenic drugs and immunotherapy remain unclear. One speculation is that TKI drugs target multiple pathways, including the VEGF/VEGFR pathway, as well as other targets related to immune regulation such as platelet-derived growth factor receptor (PDGFR), angiopoietin-2 receptor (TIE2), and colony-stimulating factor 1 receptor (CSF-1R). This broader inhibition may lead to a stronger synergistic effect on anti-angiogenesis and immunotherapy (96). Although antibody drugs targeting VEGF and EGFR in combination with immunotherapy have provided some higher-level evidence from RCTs, their clinical efficacy remains unsatisfactory. The lack of prospective controlled studies on the combination of TKI drugs and immunotherapy suggests that drawing definitive conclusions at this stage would be premature. Further research is needed to better understand the underlying mechanisms and identify optimal treatment strategies for combining TKIs with immunotherapy in the treatment of MSS mCRC.

#### Dual ICIs

Tumor cells have developed mechanisms to evade immune surveillance by inhibiting the activation and effector functions of cells in the innate and adaptive immune systems. Currently available ICIs primarily target the CD28/CTLA-4 (such as Ipilimumab) immune regulatory system or the PD-1/PD-L1 (most ICIs) interaction. “Opdivo+Yervoy” respectively play a role in the initiation and effector stages of the immune response. CTLA-4 regulates the immune response in the early stage of T cell activation, while PD-1 exerts inhibitory effects on T cell activity during the effector phase. CTLA-4 is highly expressed in activated T cells and can inhibit T cell activity. CTLA-4 inhibitors are likely to restore anti-tumor immunity and exert anti-tumor effects. PD-L1 and PD-1 play a role in immune evasion in the TME. PD-1/PD-L1 inhibitors can prevent cancer cells from escaping immune surveillance by exhausted T cells. Therefore, combining these two types of inhibitors can enhance the therapeutic effect and achieve a synergistic effect (97–99).

The CCTG CO.26 study evaluated the combination therapy of dual ICIs (Durvalumab + Tremelimumab) as salvage treatment for mCRC. The results showed that the experimental group had a significantly prolonged OS (6.6 months vs. 4.1 months) compared to the best supportive care group. However, there was no significant extension in PFS, and the response rate was only 1% (100). Subsequent analysis revealed a median tumor mutational burden (TMB) of 20.4 mt/Mb, and patients with a TMB greater than 28 mt/Mb derived more benefit. Another study, C-800, found in its expanded data that the dual immunotherapy of CTLA-4 antibody Botensilimab and PD-1 antibody Balstilimab induced objective responses in heavily pretreated MSS mCRC patients, with evidence of durability (101, 102).

This combination therapy still requires further validation through larger clinical studies. Some early studies have reported on other immune combination drugs, such as the combination of lymphocyte-activation gene 3 (LAG-3) antibody MK4280 with Pembrolizumab, and the combination of transforming growth factor-beta (TGF-β) type 1 receptor inhibitor Vactosertib with Pembrolizumab, which are still in phase I studies (103, 104). One of the main obstacles in achieving efficacy with ICIs is the limited infiltration of immune cells in the TME. In the case of MSS CRC, there is a lack of sufficient immune cells available for activation by ICIs in the TME. Furthermore, patients with high TMB are limited in clinical practice. These challenges highlight the need for further research and exploration to identify strategies that can enhance the efficacy of immunotherapy in MSS CRC. This may involve investigating novel combination therapies, identifying biomarkers that can predict response to immunotherapy, and developing approaches to increase the presence of immune cells in the TME. Larger clinical studies are needed to validate the potential of these strategies and to improve outcomes for patients with MSS CRC.

#### Immunotherapy plus others

The key to improving the efficacy of immunotherapy for MSS CRC lies in converting “cold” tumors into “hot” tumors, enhancing tumor immunogenicity, and increasing immune cell infiltration in the TME. Oncolytic virus (OV) infection can promote the release of tumor antigens, transforming the tumor from “cold” to “hot” and enhancing the anti-tumor effect of ICIs (105, 106). Therefore, combining ICIs with OVs can enhance the anti-tumor ability of ICIs.

A preliminary mid-term analysis of a phase I/II study (NCT03206073) investigating the combination of Pexa-Vec (an oncolytic vaccinia virus) with durvalumab and monotherapy with Cemiplimab for MSS mCRC showed good tolerability and no new safety concerns. However, long-term efficacy results have not yet been reported (107). Multiple clinical trials combining OVs with ICIs for MSS CRC are currently underway, such as Pexa-Vec combined with Durvalumab and TBio-6517 combined with Pembrolizumab, but most of them are in phase I studies.

In addition, some epigenetic drugs can reshape the TME, enhance the expression of tumor antigens, antigen presentation molecules, co-stimulatory molecules, and promote the infiltration of immune cells into the TME, thereby improving the efficacy of PD-1 monoclonal antibodies. In the future, exploring more strategies involving the combination of immunotherapy and drugs that reshape the TME holds promise for improving treatment outcomes in MSS CRC.

#### Immunotherapy in neoadjuvant and adjuvant therapy

There is a growing consensus in the field of tumor treatment that early application of immunotherapy can benefit patients. As a result, many researchers have explored the use of immunotherapy in neoadjuvant or adjuvant therapy during the perioperative period to prolong patients’ disease-free survival (DFS) and OS.

The NICOLE study is the first to investigate the use of the anti-PD-1 drug nivolumab as neoadjuvant therapy for unselected early-stage colon cancer with unknown MMR status. Among 44 patients with resectable colon cancer and cT3/T4 tumors, the trial arm (86% MSS) received Nivolumab monotherapy before surgery, while the control arm (77% MSS) underwent immediate surgery (108). The results showed that all 22 patients in the trial arm underwent curative resection without delayed or surgical complications. Major pathological responses (≤ 10% viable tumor cells) were observed in two pMMR/MSS tumors (including one complete response) and one pMMR/MSI-H tumor. In four confirmed MSS tumors, ≥ 30% tumor regression was observed, while no major pathological responses were seen in the two dMMR/MSI-H tumors. In the NICOLE arm, more than 70% of patients showed significant downstaging, and compared to the control arm, the NICOLE arm had significantly higher levels of CD8 and CD8 non-suppressive T cells in the tumor. Similar results have been observed in studies such as NICHE, VOLTAGE-A (109), and AVANA (110).

These studies suggest that immunotherapy is gradually demonstrating great potential and advantages in neoadjuvant and adjuvant therapy during the perioperative period. In the future, further validation through more clinical research is expected, and the findings may be incorporated into treatment guidelines.

#### Biomarkers of immunotherapy in MSS CRC

The most important approach to overcoming immunotherapy resistance in MSS CRC patients is to identify the key mechanisms of immune escape and tolerance through basic or translational research. While efforts are being made to explore the mechanisms and develop new drugs, another strategy currently being explored in clinical practice is the identification of potential molecular biomarkers of immunotherapy benefit in MSS CRC.

One such biomarker is rare DNA polymerase epsilon (POLE)/polymerase delta (POLD) gene mutations, which are recognized as predictive biomarkers for immunotherapy efficacy in MSS CRC (111). These mutations occur in approximately 7% of all CRCs, with the majority occurring in MSS CRC. POLE/POLD1 gene mutations in CRC are often associated with a high TMB. Previous studies have shown that POLE or POLD1 mutations are predictive biomarkers for effective immunotherapy in solid tumors (112). Subsequent retrospective study results further suggest that the presence of pathogenic POLE mutations may be a key factor for immunotherapy sensitivity (113).

Apart from these biomarkers, there are currently no other recognized predictive biomarkers or clinical pathological features for immunotherapy efficacy in MSS CRC, including PD-L1 and TMB, which are recognized in other tumor types. While high expression of PD-L1 in tumor tissue is associated with immunotherapy efficacy in many solid tumors, prospective studies in advanced CRC have shown that its expression is not predictive of immunotherapy efficacy (114). Although subsequent analysis of the CCTG CO.26 study showed a correlation between TMB and the efficacy of dual immunotherapy, the optimal cut-off value for TMB and its clinical significance remain controversial (62, 115, 116).

In addition, subgroup analyses of multiple studies have suggested that liver metastasis is a negative factor for immunotherapy efficacy in CRC. Liver metastases have shown minimal response to immunotherapy, possibly due to the induction of CD8+ T cell apoptosis or dendritic cell dysfunction by macrophage subpopulations in the liver immune microenvironment (117, 118). Tumor lymphocyte infiltration, especially the density of CD8+ T cells, is associated with the prognosis of CRC (119). Immune scoring based on the proportion of tumor immune cells has shown higher predictive value for prognosis in early and middle-stage CRC compared to TNM staging and MSI status (120). However, the predictive value of immune scoring for immunotherapy efficacy in advanced CRC has not yet been demonstrated.

## Chimeric antigen receptor T cell therapy

Chimeric antigen receptor T (CAR-T) cell therapy is an innovative form of genetically engineered immunotherapy that has shown significant progress in the treatment of certain tumors, particularly those of the hematopoietic system (121). However, its effectiveness in solid tumors, including CRC, is not as ideal as in hematopoietic tumors (122–124).

T cells play a crucial role in eliminating tumor cells in the human body. In CAR-T cell therapy, T cells are obtained from the patient’s own blood or from a healthy donor’s blood and genetically modified using gene engineering techniques to express chimeric antigen receptors (CARs) that specifically recognize tumor antigens. By combining the antigen-antibody binding mechanism with the cytotoxic effect of T cells, CAR-T cells acquire the ability to target and destroy tumor cells (124). CAR-T cell therapy has now advanced to the fourth generation (125).

The key to successful CAR-T cell therapy for tumors lies in identifying specific target antigens. Tumor-associated antigens (TAAs) are expressed to some extent in normal tissues but are overexpressed in tumor tissues, making them easily recognizable by CAR-T cells. Common targets for CRC include EpCAM, carcino-embryonic antigen (CEA), epidermal growth factor receptor (EGFR), placental alkaline phosphatase (PLAP), human epidermal growth factor receptor-2 (HER-2), tumor-associated glycoprotein-72 (TAG-72), guanylate cyclase C (GUCY2C), Natural killer group 2 member D (NKG2D), and others.

CAR-T cell therapy has transitioned from preclinical experiments to clinical research. However, the results of CAR-T cell therapy for solid tumors have shown limited efficacy and poorer treatment responses, primarily due to inappropriate antigens, on-target, off-tumor toxicities, and limited tumor cell infiltration (124, 126, 127). Therefore, current research focuses on finding suitable antigens and increasing T cell infiltration.

In the case of MSS CRC, the TME tends to be “cold” and lacks T-cell inflammation, which limits the functionality of CAR-T cells. Various approaches are being explored to modify the TME and increase T cell infiltration, thereby transforming tumors from an immune “cold” state to an immune “hot” state through various mechanisms (128). Preliminary data on CAR-T cell therapy combined with chemotherapy, radiation therapy, or targeted therapy provide promising evidence for combination treatments and warrant further investigation (129, 130).

## Cancer vaccine in MSS CRC

Cancer vaccines have emerged as a promising area of research in recent years and are considered a form of active immunotherapy. The principle behind cancer vaccines involves introducing tumor antigens into the patient’s body to overcome immune suppression caused by tumors, enhance immunogenicity, activate the patient’s own immune system, induce cellular and humoral immune responses, and ultimately control or eliminate tumors (131).

There are several types of cancer vaccines, including whole-cell tumor vaccines, dendritic cell vaccines, peptide vaccines, and nucleic acid vaccines. DCs are known for their strong antigen-presenting function and are often referred to as the “sentinels” of the immune system. They play a crucial role in initiating immune responses against tumor antigens. However, tumor-infiltrating DCs are often scarce and functionally impaired in the host’s body. Therefore, a strategy that has shown effectiveness is to culture host DCs and load them with tumor antigens to prepare DC tumor vaccines. In April 2010, the U.S. FDA approved the first autologous cellular immunotherapy drug, sipuleucel-T (Provenge), which primarily consists of DCs, for the treatment of asymptomatic or minimally symptomatic metastatic castration-resistant prostate cancer (132). More recently, personalized dendritic cell vaccines have demonstrated excellent efficacy in newly diagnosed glioblastoma (nGBM) and recurrent glioblastoma (rGBM) patients, significantly prolonging patient survival (133).

A phase I/II study (NCT03639714) is currently underway to evaluate the safety, tolerability, and recommended phase II dose (RP2D) of an individualized heterologous chimpanzee adenovirus (ChAd68) and self-amplifying mRNA (samRNA)-based neoantigen vaccine in combination with Nivolumab and Ipilimumab in patients with advanced metastatic solid tumors. Preliminary results from this study have shown improved OS and reduced circulating tumor DNA (ctDNA) in several patients with MSS-CRC, indicating good patient tolerance and no dose-limiting toxicities (134). These findings support the need for larger randomized clinical trials. A randomized phase II/III study (NCT05141721) has been initiated to demonstrate the efficacy of this vaccine regimen in patients with MSS CRC in the first-line metastatic setting.

On the other hand, a phase II clinical study using whole-cell cancer immunotherapy as a vaccine in patients with MSS CRC showed no objective responses (NCT02981524). This limitation could be attributed to inappropriate antigen selection, adjuvant selection, vaccine platform, and/or delivery methods.

Vaccines have the ability to activate CTLs, and there is a significant correlation between infiltrating lymphocytes and OS in patients with MSS disease (135). Combining vaccines with anti-PD-1/PD-L1 treatment may provide new insights for refractory MSS CRC patients (136).

## Perspectives

The colon is a complex environment that contributes to the complexity of CRC. Despite the increasing understanding of the TME and its role in CRC, there are still many unknown areas to explore, particularly considering the intricate intestinal environment and the constantly changing microbiota. The majority of mCRC cases are MSS, and due to the presence of an immunosuppressive microenvironment, MSS CRC has lower responsiveness to single-agent immunotherapy. Therefore, it is crucial to elucidate the characteristics of the CRC microenvironment to develop novel therapeutic approaches.

There is still uncertainty regarding which cells interact closely in the TME, how they interact with each other, and how the TME influences these interactions. Additionally, the tumor-associated microbiota is an intrinsic component of the TME in various human cancer types. However, current studies on the host-microbiota interaction within tumors primarily rely on bulk tissue analysis, which may mask the spatial distribution and local effects of the tumor microbiota. Therefore, integrating single-cell transcriptomic data with high-dimensional spatial transcriptomic data would be beneficial in understanding the spatial and interaction dynamics between the host, microbiota, and TME cells. This integration would be crucial in elucidating the TME (29).

There have been numerous studies exploring colon cancer tumors, highlighting the need to explore new treatment approaches for MSS CRC. MSS CRC, characterized as a “cold tumor” insensitive to ICIs, presents challenges and progresses slowly in the field of immunotherapy. However, several small-sample studies have preliminarily explored various immune combination therapy strategies, showing promising prospects in the neoadjuvant treatment of advanced and locally advanced CRC. Tumor vaccines have not made significant progress in recent years, but the combination of new antigen vaccines based on samRNA and immunotherapy has demonstrated a role in prolonging OS in MSS CRC, generating anticipation for the results of phase II/III clinical trials investigating this combination therapy. Furthermore, exploring the combination of CAR-T cell therapy with chemotherapy, radiotherapy, or targeted therapy holds promise and warrants further investigation.

**Figure 2:**
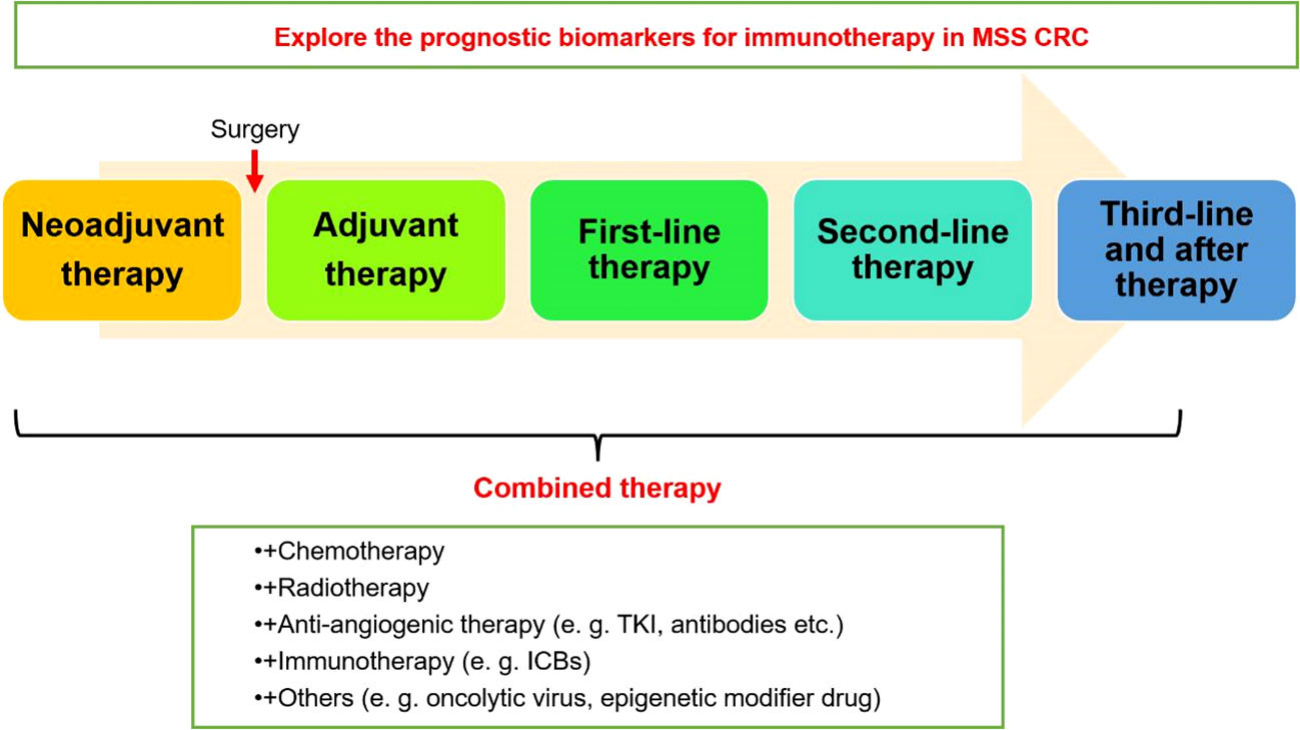
Clinical application strategies of immunotherapy in MSS CRC.

Future breakthroughs in immunotherapy for MSS CRC depend on the collaborative efforts of basic and clinical researchers to elucidate the key mechanisms and targets of immune evasion and immune tolerance in MSS CRC. This knowledge will guide the development of more effective immunotherapy approaches or drugs. Currently, expanding the sample size of RCTs is necessary to consolidate the effectiveness of immunotherapy combined with other treatments. Additionally, further exploration and identification of biomarkers are needed to guide the selection of immunotherapy beneficiaries and emphasize effective treatments for specific individuals, guiding the next stage of more precise research and treatment strategies (**Figure 2**). The prospects for immunotherapy in MSS CRC are promising.

## Author contributions

KD: Writing – original draft. PM: Funding acquisition, Writing – original draft. ZW: Writing – original draft. SL: Writing – original draft. JL: Funding acquisition, Supervision, Writing – review & editing. HL: Supervision, Writing – review & editing. GY: Supervision, Writing – original draft, Writing – review & editing.

## Glossary

**Table:**

CRC	colorectal cancer
ICIs	immune checkpoint inhibitors
dMMR	deficient mismatch repair
MSI-H	high microsatellite instability
pMMR	proficient mismatch repair
MSS	microsatellite stability
TME	tumor microenvironment
MLH1	mutL homolog 1
MSH2	mutS homolog 2
MSH6	mutS homolog 6
PMS2	postmeiotic segregation increased 2
mCRC	metastatic CRC
CTLA-4	cytotoxic T lymphocyte-associated antigen-4
TAM	tumor-associated macrophages
DC	dendritic cells
NK	natural killer
TILs	tumor-infiltrating lymphocytes
CTLs	cytotoxic T cells
Tregs	regulatory T cells
HLA-G	human leucocyte antigen-G
SPP1	secreted phosphoprotein 1
CAFs	cancer-associated fibroblasts
MSCs	mesenchymal stem cells
ILCs	innate lymphoid cells
MHC-I	major histocompatibility complex class I
ORR	objective response rate
DCR	disease control rate
OS	overall survival
VEGF	vascular endothelial growth factor
TKIs	tyrosine kinase inhibitors
RCT	randomized controlled trials
PFS	progression-free survival
CAR-T	chimeric antigen receptor T
